# Segmental areas of denervation in post-polio syndrome

**DOI:** 10.1055/s-0044-1779507

**Published:** 2024-02-23

**Authors:** Vinícius Lopes Braga, Heloísa Lopes Cohim Moreira, Pedro Henrique Almeida Fraiman, Filipe Pereira Sarmento, Igor Braga Farias, Paulo de Lima Serrano, Bruno de Mattos Lombardi Badia, Marco Antônio Troccoli Chieia, Wladimir Bocca Vieira de Rezende Pinto, Paulo Victor Sgobbi de Souza, Acary Souza Bulle Oliveira

**Affiliations:** 1 Universidade Federal de São Paulo, Escola Paulista de Medicina, Departamento de Neurologia e Neurocirurgia, São Paulo SP, Brazil.

**Figure 1:**
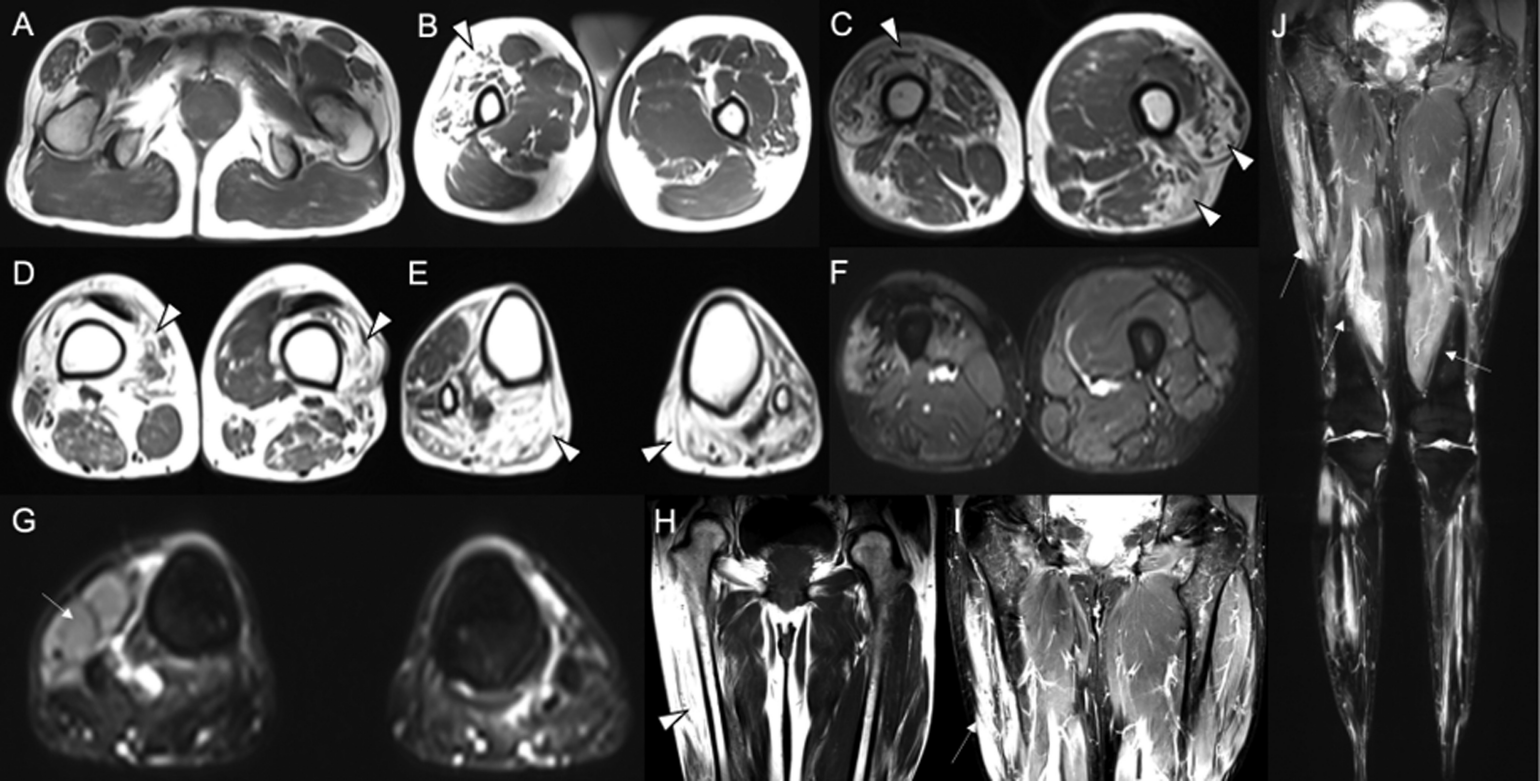
Muscle MRI studies. Axial ( **A-E** ) and coronal ( **H** ) T1-weighted MRI shows asymmetric amyotrophy and marked fatty replacement involving bilateral vastus lateralis, right vastus intermedius, left biceps femoris, right vastus medialis, left tibialis anterior, and bilateral heads of gastrocnemius. Axial ( **F,G** ) and coronal ( **I,J** ) STIR MRI shows hyperintensity involving right vastus lateralis and tibialis anterior.

A one-year-old male disclosed motor developmental delay, appendicular hypotonia, hyporeflexia, and weakness in the left lower limb. He started walking only at three years old. After this time, his symptoms were stable. At 31 years old, the patient started slight asymmetric and slowly progressive paraparesis and myalgia. He was referred with a suspicion of spinal muscular atrophy. Electromyography disclosed bilateral chronic lumbosacral denervation and mild acute denervation involving the right lower limb. Neuroimaging studies (brain and spinal MRI) and genetic testing (MLPA test for quantification of SMN1 and SMN2 copy numbers and hereditary neuropathy, non-5q, and other motor neuron disease panels) were normal. Muscle MRI disclosed asymmetric neurogenic muscle “islands” alternating areas with and without fatty replacement ( Figure 1 ), 1 suggestive of Post-Poliomyelitis Syndrome (PPS). 2 3 Muscle MRI is useful to distinguish PPS from other motor neuron diseases. 1 2 3
